# Experimental verification of the proton range using heterogeneous animal tissue

**DOI:** 10.1002/acm2.70654

**Published:** 2026-06-01

**Authors:** Andrew J. Boria, Chia‐Ho Hua, Thomas E. Merchant, Chin‐Cheng Chen

**Affiliations:** ^1^ Department of Radiation Oncology St. Jude Children's Research Hospital Memphis Tennessee USA

**Keywords:** animal tissue, proton, proton range, radiation oncology, radiotherapy, range uncertainty

## Abstract

**Background:**

Proton beam therapy offers comparable tumor control to conventional photon therapy while reducing radiation‐related toxicity due to its finite range and minimal exit dose beyond the Bragg peak. However, the accuracy of proton dose delivery is highly dependent on precise knowledge of tissue stopping power along the beam path. Small deviations in tissue composition or density can result in clinically meaningful proton range errors, potentially compromising target coverage and normal tissue sparing. A primary contributor to this uncertainty is the conversion of computed tomography (CT) Hounsfield Units (HU) to relative stopping power using a bilinear calibration curve, which provides only an approximation of patient tissue properties. Although robustness optimization strategies in modern spot‐scanning proton therapy help mitigate the dosimetric impact of range uncertainty, uncertainties in HU‐to‐stopping‐power calibration remain a fundamental limitation of CT‐based proton treatment planning. Consequently, there is an ongoing need for practical, efficient methods to verify and validate bilinear calibration curves under clinical conditions, ensuring accurate modeling of proton range and distal dose falloff while maintaining compatibility with routine quality assurance workflows.

**Purpose:**

To present an end‐to‐end test for verifying proton range calculations derived from a recently established CT bilinear calibration curve, using animal tissues and standard QA tools. This study provides a robust framework for preclinical verification of proton dose calculations in complex, heterogeneous tissues and supports the safe delivery of proton therapy.

**Methods:**

A CT calibration curve (mass density vs. Hounsfield units) was generated for the RayStation Treatment Planning System 2023B (RaySearch Laboratories, Stockholm, Sweden) using a stoichiometric method. A 20×20×10 cm^3^ PMMA container was filled with combinations of animal muscle, adipose, and bone tissues to simulate heterogeneous anatomy. Proton treatment plans were optimized with RayStation's Monte Carlo (MC) algorithm to deliver a uniform, single‐fraction dose of 200 cGy(RBE). Range accuracy was assessed by measuring 2D planar dose distributions at multiple depths with an ionization chamber array. Proton range parameters including R90, R80, R50, and R20 were determined from measured depth‐dose curves and compared with the range parameters calculated by both MC and Pencil Beam Convolution Superposition (PBCS) algorithms.

**Results:**

Measured and calculated range parameters showed excellent agreement across all tissue configurations. Regarding R80, for the first three scenarios, percentage differences were noticeably minimal: 0.24% (MC) and 0.16% (PBCS) for a water‐equivalent thickness (WET) of 18.3 cm (muscle/adipose); 0.47% (MC) and 0.47% (PBCS) for 10.7 cm (muscle/adipose) plus 3.5 cm (bone); and 0.65% (MC) and 0.56% (PBCS) for 9.4 cm (muscle/adipose) plus 4.7 cm (bone). Even in the most challenging configuration, with 3 cm of lateral heterogeneity (bone intersecting muscle+adipose) plus 14.3 cm of muscle/adipose, differences remained low at 1.21% (MC) and 1.08% (PBCS).

**Conclusions:**

This end‐to‐end method provides an effective approach for verifying proton range calculations in heterogeneous tissues, supporting accurate and robust treatment planning. This approach is especially valuable when implementing proton treatment planning systems, providing increased confidence in the reliability of proton therapy delivery.

## 1 INTRODUCTION

Proton beam therapy matches the effectiveness of photon therapy but with lower toxicity because of minimal exit dose past the Bragg peak.^1^, ^2^, ^3^ However, proton range is sensitive to tissue stopping power, so small variations in tissue composition can shift dose distributions. This consequentially affects both target coverage and normal tissue sparing.^4^, ^5^, ^6^

A major challenge is range uncertainty, primarily from the inexact conversion of Hounsfield Units (HU) to relative proton stopping powers (RSP) via a CT‐based bilinear calibration curve. This leads to range uncertainty, which can impact treatment accuracy.^7^, ^8^, ^9^ While robustness optimization in spot‐scanning proton therapy mitigates the effects of range shifts,^10^, ^11^, ^12^ accurate HU‐to‐RSP calibration remains critical to ensure CT‐based estimates reflect tissue properties during treatment.

To address this, we developed a streamlined end‐to‐end method to validate the CT bilinear calibration curve used in proton dose calculations. It uses common QA tools, such as ion‐chamber arrays, to measure proton range, avoiding cumbersome multilayer ionization chambers (MLICs). Unlike MLICs, easily deployed ion‐chamber arrays can assess range along multiple beam paths in a single delivery and allow rapid depth adjustments, greatly increasing efficiency and accessibility. When implemented within a commercial treatment planning system, the proposed method demonstrates accurate modeling of distal dose falloff and constitutes a meaningful advancement toward more reliable proton therapy delivery using readily accessible and straightforward clinical equipment.

## 2 METHODS

### 2.1 CT calibration curve

A CT calibration curve relating Hounsfield units to proton stopping power was generated using the stoichiometric method of Schneider et al.^13^ and implemented in RayStation TPS 2023B (RaySearch Laboratories, Stockholm, Sweden) using scans from a Philips IQon Spectral CT (Philips Healthcare, Cleveland, Ohio). The method involves scanning tissue‐equivalent samples to correlate CT HUs with mass density for proton dose calculations. Calibration data were acquired using an in‐house protocol with a CIRS Brain Constancy exam card (120 kVp, 500 mAs, 16 × 0.625 mm collimation, UB filter, 200 mm FOV, 1 mm slices, iDose(3)) and a Body Constancy exam card (120 kVp, 400 mAs, 16 × 0.625 mm collimation, Standard B filter, 370 mm FOV, 1.5 mm slices, iDose(3)).

CT model equations were developed by analyzing CT numbers and compositions of tissue‐equivalent samples from dual head/body phantoms. Using known ICRU tissue compositions, predicted CT numbers were calculated for each tissue under each scan condition, and a piecewise linear curve was fitted to predicted HU versus known physical density. Final calibration curves for each scanner were obtained by averaging the head and body curves.

**FIGURE 1:**
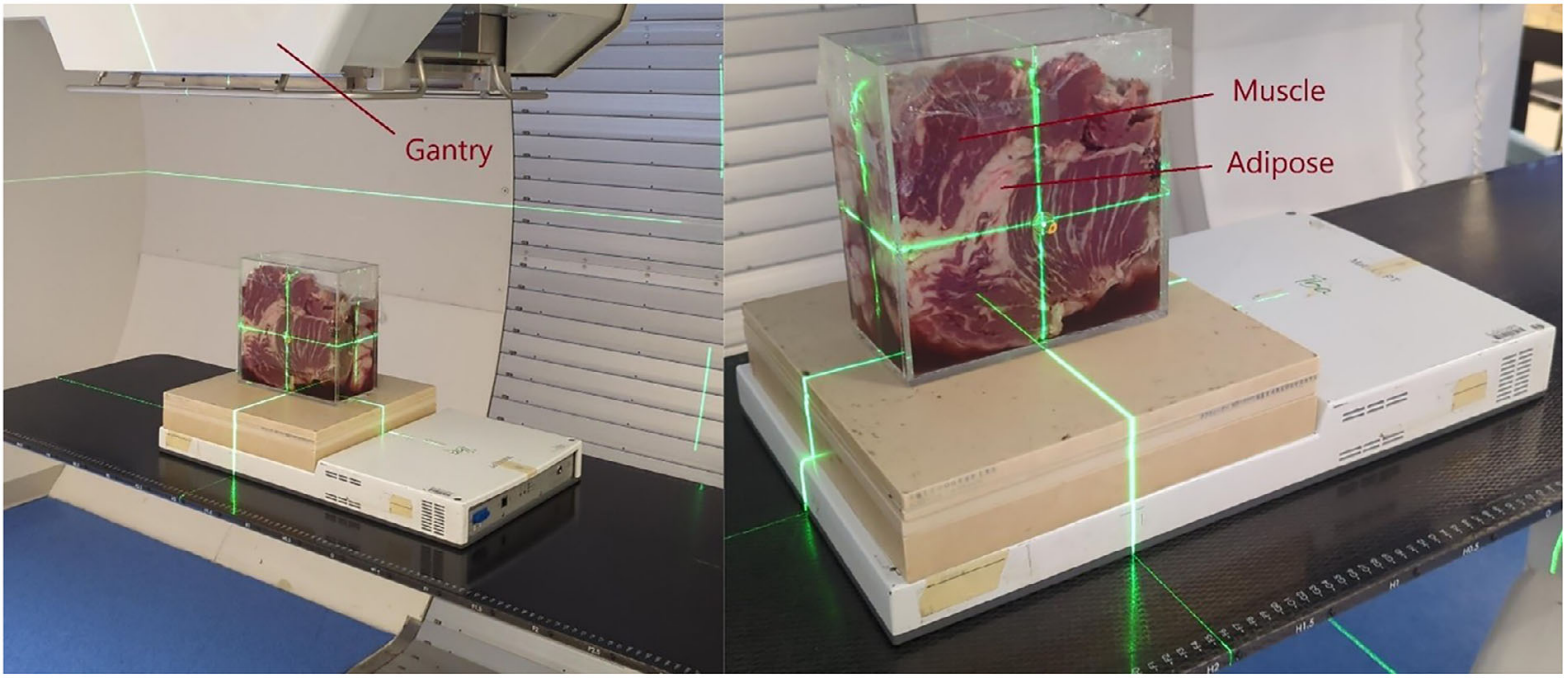
Treatment setup from two views. Tough water thickness and source‐to‐surface distance (SSD) were varied to adjust measurement depth. PROTON nozzle, muscle tissue, and adipose tissue are labeled.

After generating the CT calibration curve, a 20 × 20 × 10 cm^3^ PMMA container (with a commissioning validated PMMA override) was filled with bovine muscle, adipose, and bone tissues arranged in beam path configurations of increasing complexity (soft tissue → bone → lateral heterogeneities). Tissue thicknesses were chosen to span clinically relevant water equivalent depths typical of pediatric and adult proton therapy (approximately 15–25 cm), while remaining compatible with the fixed container dimensions. These configurations were designed to represent clinically relevant range scenarios rather than a specific patient anatomy. The filled container and treatment plan layout used for phantom irradiation with an IBA MatriXX PT 2D ionization chamber array (IBA Dosimetry, Schwarzenbruck, Germany) are shown in Figure 1.

### 2.2 Treatment field

**FIGURE 2:**
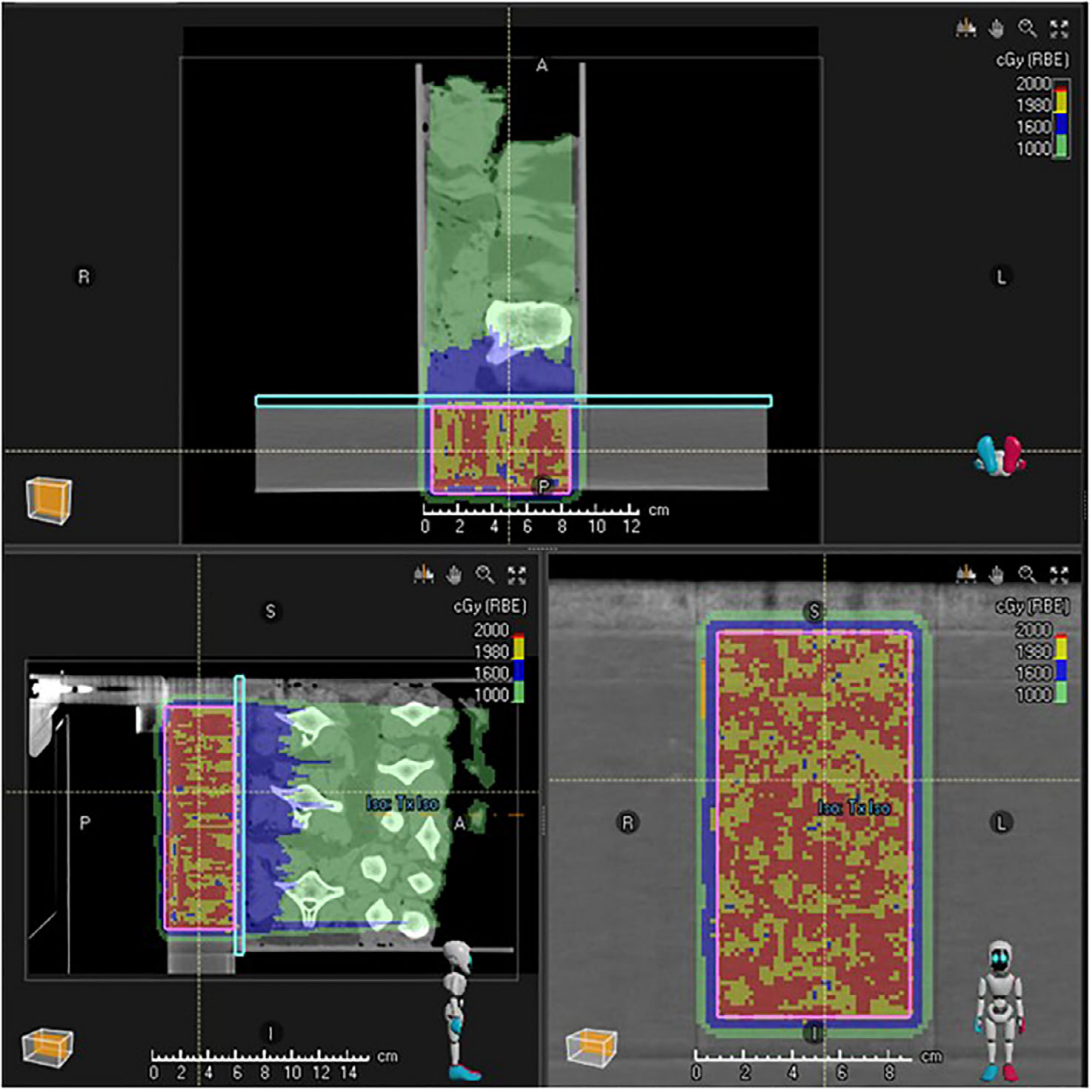
Animal tissue phantom and dose distribution (2 mm grid, discrete color wash) for a single anterior‐to‐posterior proton field. The plan delivers 200 cGy(RBE) to the CTV beneath the phantom.

A pencil‑beam scanning proton plan with a single anteroposterior beam (144.2–200.4 MeV; hexagonal spot placement and automatic energy‑layer and spot spacing) was optimized using RayStation's Monte Carlo (MC) algorithm (0.2% uncertainty), with robust optimization intentionally disabled to prevent concealment of range calculation errors, was designed to deliver a uniform dose of 200 cGy(RBE) within an 8 × 16 × 5 cm^3^ volume beneath the phantom, as shown in Figure 2. Laser alignment to the BBs on the phantom at the plan isocenter, followed by CBCT, was used to align the phantom to simulation conditions. A consistent isocenter was maintained for all measurements. The 2D planar doses were measured from the ion chamber in the array using a best fit approach between the expected and measured dose distributions, using the MatriXX PT two‑dimensional ionization chamber array at multiple depths, realized with Tough Water (PH‐40) phantom slabs (Kyoto Kagaku Co., Ltd., Kyoto, Japan). We assumed a relative stopping power (RSP) of 1.00 ± 0.01 for PH‐40 based on tabulated density and electron‐density data. This assumption was further evaluated using MLIC measurements of Tough Water at the median plan energy in two configurations: one without additional Tough Water and one with an added 5 cm layer of Tough Water. These measurements yielded an RSP of approximately 1.004 and provide baseline support for the proposed method, which determines range parameters by taking measurements with varying thicknesses of solid water positioned beneath the animal phantom.

Proton range parameters, defined in this study as R90, R80, R50, and R20, represent the depths in water at which the dose falls to 90%, 80%, 50%, and 20% of the intended delivered dose at the peak of the spread‑out Bragg peak (SOBP), respectively. These parameters were determined from depth–dose curves, which were also evaluated for discrepancies. Among these metrics, R80 is the most useful for commissioning, as it is commonly employed to define the nominal range and provides a more reproducible and robust reference point, particularly when analyzing measured data affected by detector noise, scanning uncertainties, and upstream ripple.^14^ Furthermore, studies have shown that R80 is a good indicator of the mean energy of the beam spectrum and is independent of variations in the initial energy spread, even between different devices.^15^ The measured results were compared with the doses calculated by MC as well as (separately recalculated) PBCS. A constant relative biological effectiveness of 1.1 was used for proton beams.

### 2.3 Beam paths & tissue combinations

**FIGURE 3:**
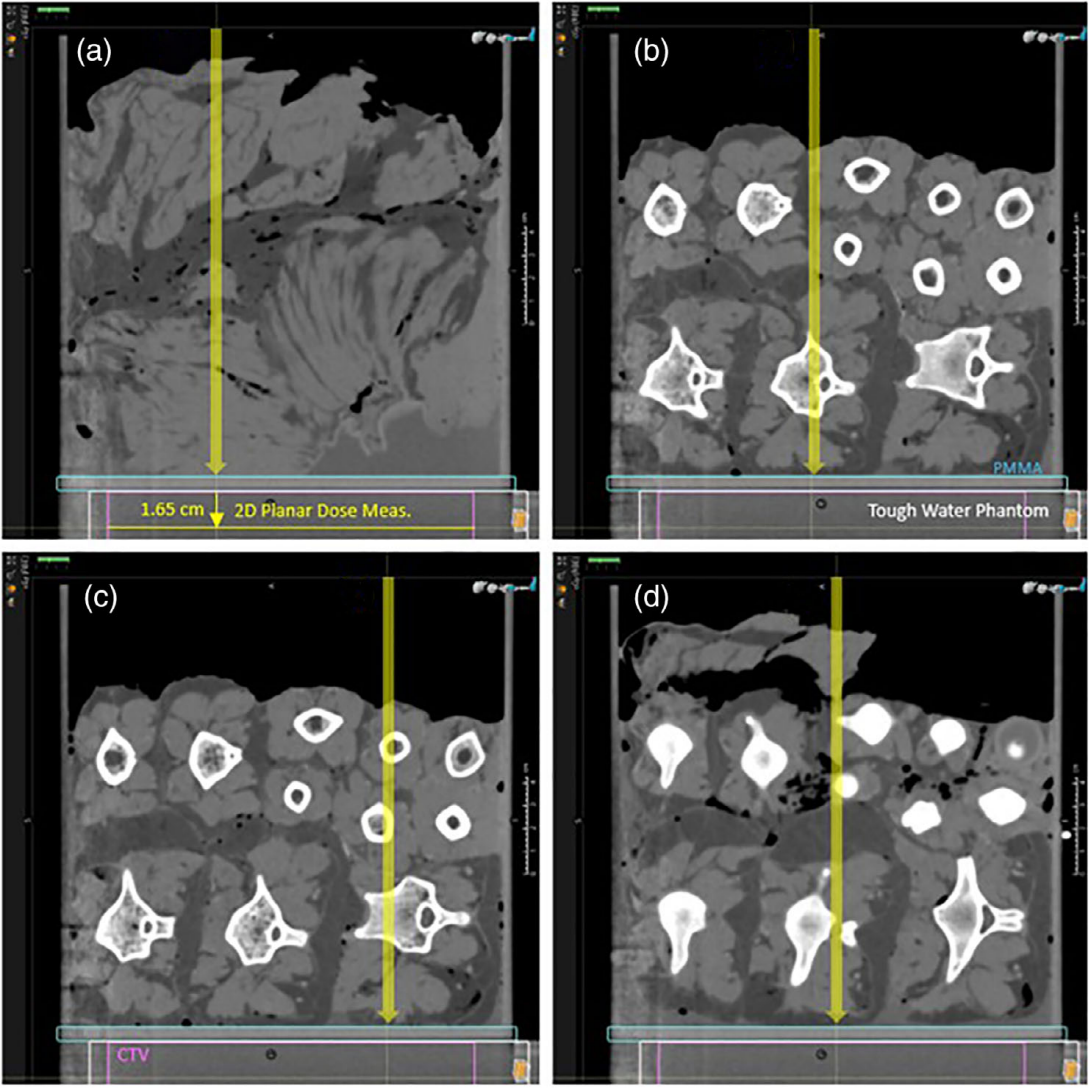
2D planar doses were measured at depths from 1.65–6.35 cm inferior to the PMMA container bottom. Point doses along beam paths A–D were compared with calculations: (a) 18.3 cm muscle+adipose. (b) 10.7 cm muscle+adipose + 3.5 cm bone. (c) 9.4 cm muscle+adipose + 4.7 cm bone. (d) 3 cm lateral heterogeneity (bone intersecting muscle+adipose) + 14.3 cm muscle+adipose.

Distinct tissue combinations along beam paths through the phantom were analyzed at ten depths (1.65–6.35 cm) to assess tissue heterogeneity effects (Figure 3):

- A: Homogeneous soft tissue; 18.3 cm muscle/adipose
- B: Soft tissue and bone; 10.7 cm muscle/adipose + 3.5 cm bone
- C: Soft tissue and bone; 9.4 cm muscle/adipose + 4.7 cm bone
- D: Lateral heterogeneity; 3 cm mixed bone and soft tissue + 14.3 cm muscle/adipose

## 3 RESULTS

### 3.1 Measured vs calculated depth‐dose curve analysis

**FIGURE 4:**
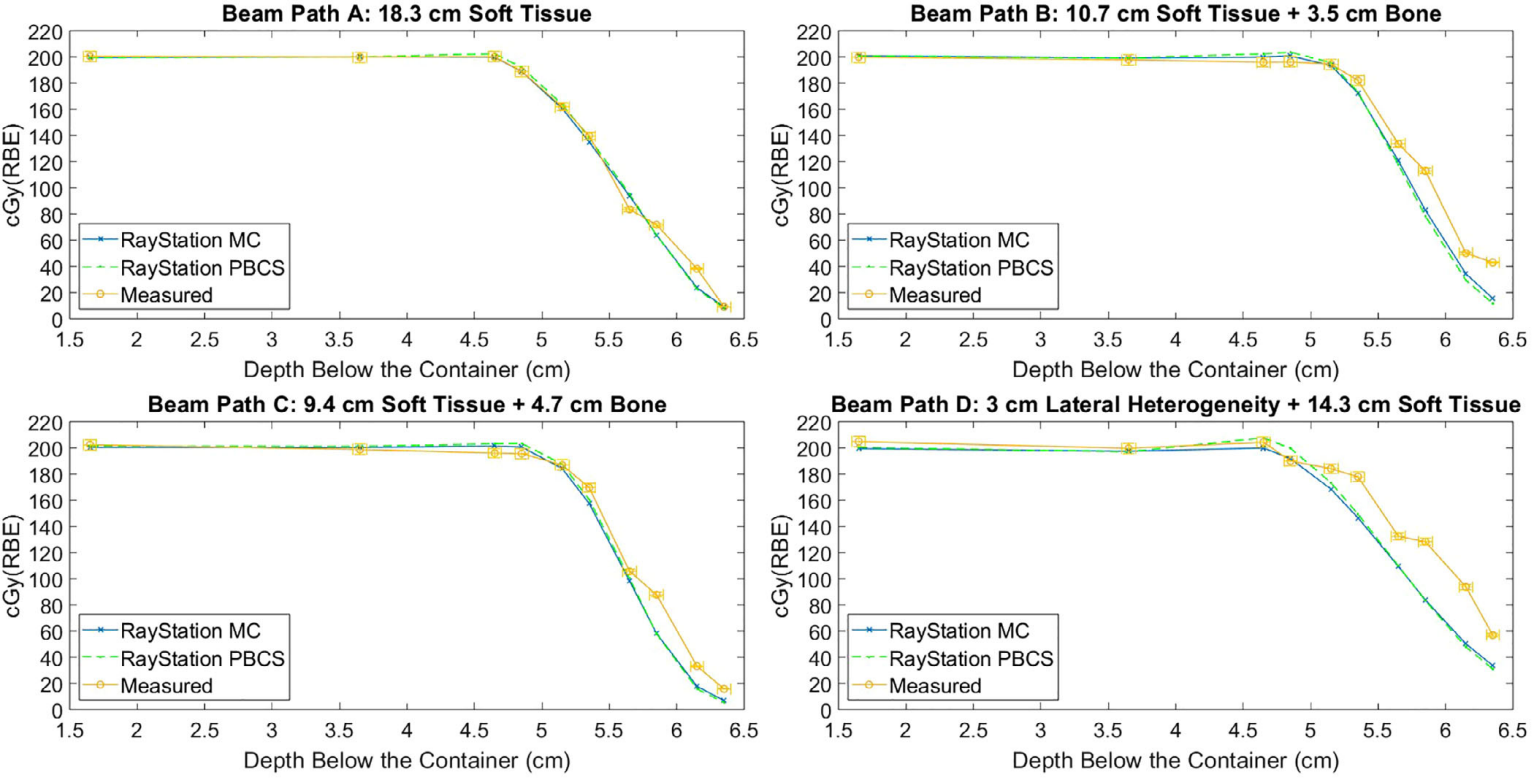
Dose vs depth for RayStation MC, PBCS, and measured data for Beam Paths A–D. Depths on the x‐axis include Tough Water plus 0.65 cm WET of the IBA MatriXX PT device. Beam path materials are the same as in **Figure** **3**.

Figure 3 illustrates the tissue combinations for Beam Paths A–D, while Figure 4 shows the corresponding measured and calculated depth‐doses. The closest agreement is observed in Beam Path A (muscle+adipose tissue). Beam Paths B and C, which include bone, show slightly greater discrepancies, particularly near the end of the proton range. The largest divergence occurs in Beam Path D, involving a lateral heterogeneity, where deviations emerge as the dose falls from the SOBP peak. Below 90% of the maximum dose, differences range from ∼20–45 cGy(RBE) (∼2–4 mm), with a maximum deviation of ∼4 mm between 128.26 and 93.87 cGy(RBE), which decreases near the end of range to ∼25 cGy(RBE) (∼2.5 mm).

Figure 4 shows minimal differences between RayStation's MC and PBCS algorithms beyond the SOBP. Beam Path A exhibits no discernible difference, while Beam Paths B and C show only minor divergences. In contrast, Beam Path D (lateral heterogeneity) demonstrates noticeable deviations both just before the post‐SOBP dose falloff and around 25 cGy(RBE). The largest divergence occurs immediately prior to dose falloff, at a WET of 4.65 cm, where MC and PBCS doses are 199.9 and 207.7 cGy(RBE), respectively.

### 3.2 Proton range parameter analysis

For proton range, defined as R80, excellent agreement was observed between calculated and measured data across all beam paths (Table 1 and Table 2) show excellent agreement, with a clear pattern of increasing divergence as transitioning from soft tissue to tissue and bone to lateral heterogeneities. Beam Path A (18.3 cm muscle/adipose) exhibited the smallest R80 differences between RayStation's MC and PBCS algorithms and measurements (0.24% and 0.16%, respectively). Beam Path B (10.7 cm muscle/adipose + 3.5 cm bone) demonstrated slightly larger but still low differences (0.47% for both MC and PBCS). Beam Path C (9.4 cm muscle/adipose + 4.7 cm bone) showed modestly increased discrepancies (0.65% MC vs measured and 0.56% PBCS vs measured). The largest R80 differences occurred for Beam Path D (3 cm lateral bone/muscle heterogeneity + 14.3 cm muscle/adipose), with differences of 1.21% and 1.08% for MC and PBCS, respectively.

**TABLE 1:** The RayStation MC and PBCS R90, R80, R50, and R20 range parameter values, along with the corresponding measured values, are presented by beam path (Figure 4). These range parameters represent the total water‑equivalent thickness (WET) from beam entry through the animal tissue, PMMA base, tough water, and the inherent 0.65 cm WET of the IBA MatriXX PT device.

Beam path	Range parameter	RayStation MC (cm)	RayStation PBCS (cm)	Measured (cm)
A	R90	24.78	24.81	24.76±0.05
	R80	24.97	24.99	25.03±0.06
	R50	25.43	25.44	25.38±0.05
	R20	25.84	25.83	25.95±0.05
B	R90	21.16	21.17	21.24±0.05
	R80	21.30	21.30	21.40±0.06
	R50	21.61	21.59	21.76±0.05
	R20	21.95	21.92	22.27±0.08^*^
C	R90	21.37	21.39	21.41±0.05
	R80	21.51	21.53	21.65±0.05
	R50	21.87	21.88	21.95±0.05
	R20	22.21	22.20	22.36±0.05
D	R90	23.81	23.88	24.07±0.09
	R80	24.02	24.05	24.31±0.06
	R50	24.51	24.51	24.89±0.05
	R20	25.06	25.03	25.23±0.08^*^

*Derived from extrapolated data

**TABLE 2:** Percentage differences (% Diff) for the four range parameters (R90, R80, R50, and R20) among RayStation MC, RayStation PBCS, and measured values, sorted by range parameter and then by beam path, as shown in Figure 4 to facilitate comparison across beam paths.

Range parameter	Beam path	% Diff: RayStation MC vs measured	% Diff: RayStation PBCS vs measured	% Diff: RayStation MC vs PBCS
R90	A	0.08%	0.20%	0.12%
	B	0.38%	0.33%	0.05%
	C	0.19%	0.09%	0.09%
	D	1.09%	0.80%	0.29%
R80	A	0.24%	0.16%	0.08%
	B	0.47%	0.47%	0.00%
	C	0.65%	0.56%	0.09%
	D	1.21%	1.08%	0.12%
R50	A	0.20%	0.24%	0.04%
	B	0.69%	0.79%	0.09%
	C	0.37%	0.32%	0.05%
	D	1.55%	1.55%	0.00%
R20	A	0.43%	0.46%	0.04%
	B	1.46%^*^	1.60%^*^	0.14%
	C	0.68%	0.72%	0.05%
	D	0.68%^*^	0.80%^*^	0.12%

*Derived from extrapolated data

Looking at Table 2, differences between RayStation MC and PBCS calculations for R80 were minimal across all beam paths. Beam Path B exhibited no difference (0%), while Beam Paths A and C showed differences of less than 0.1%. The maximum MC–PBCS difference was 0.12% for Beam Path D, corresponding to the lateral heterogeneity of tissue and bone.

Similar trends were observed for R90, with excellent agreement between calculated and measured values and minimal differences between MC and PBCS. For calculated versus measured comparisons, Beam Path A demonstrated the lowest divergence (0.08% MC vs measured and 0.20% PBCS vs measured), whereas Beam Path D showed the largest divergence (1.09% MC vs measured and 0.80% PBCS vs measured). Differences between MC and PBCS for R90 were small for all beam paths, with a maximum of 0.29% observed for Beam Path D.

Results for R50 followed trends consistent with those of R80 and R90. Agreement between calculated and measured values was high overall, with Beam Path A exhibiting the smallest differences (0.20% MC vs measured and 0.24% PBCS vs measured) and Beam Path D exhibiting the largest differences (1.55% for both MC vs. measured and PBCS vs. measured). Differences between MC and PBCS calculations for R50 remained minimal, with all percentage differences below 0.1%.

R20, representative of the tail end of distal dose falloff, used data extrapolation to acquire its R20 values for Beam Paths B and D. The final measured datapoint in Beam Path B in particular diverging at the end is the reason Beam Path B's R20 value shows the greatest divergence (1.46% MC vs. measured and 1.60% PBCS vs. measured). Otherwise, Beam Path A's % Diff MC vs. measured and PBCS vs. measured numbers are the largest of the Beam Path A four parameters at 0.43% and 0.46% respectively. Beam Path C and D very similar % Diff numbers (0.68% for both Beam Path C and D's RayStation MC vs. Measured as well as 0.72% for Beam Path C's PBCS vs. Measured and 0.80% for Beam Path D's PBCS vs. Measured). Differences between MC and PBCS results are minimal, with all four % Diff values < 0.15%.

## 4 DISCUSSION

This work presents a practical method for proton range verification using a 2D ion chamber array and Tough Water in an end‐to‐end test incorporating animal tissue. Table 2 shows that the simplest setup (Beam Path A) generally yields the lowest percentage differences across all range parameters between calculated and measured results, whereas incorporating bone content (Beam Paths B and C) and lateral heterogeneities (Beam Path D) result in larger discrepancies. Bone presents greater complexity and uncertainty in HU assignment than muscle or adipose tissue. Although often cited at approximately 1000 HU, bone is highly heterogeneous, comprising compact bone (≈600–2200 HU) and spongy bone (≈150–650 HU) with differing radiological properties. This variability increases calibration uncertainty, particularly in lateral heterogeneous geometries that combine bone, soft tissue, and complex beam paths.

Positional setup errors possibly contributed to the greater disagreement observed in Figure 4 for the lateral heterogeneity case (Beam Path D). In contrast, Beam Paths A–C are less sensitive, as minor shifts do not significantly change material composition. For Beam Path D, the beam lies at the bone–soft tissue interface, so even small lateral shifts can substantially alter the proportion of each tissue in the path. Although CBCT imaging prior to irradiation did not reveal any noticeable differences, tissue position may also change slightly during transport from the CT simulator to the proton gantry or due to decay over the ∼1 hour between imaging and irradiation, despite phantom immobilization. CBCT/kV alignment proceeding Tough Water adjustment and/or placing the Tough Water on top of the cube, eliminating the need for repeated repositioning, may further improve results in future studies.

Despite opportunities for further improvement, even in the generally worst‑case scenario (Beam Path D), the observed divergence was minimal. Maximum deviations reached approximately 0.4 cm, corresponding to a 0.38 cm R50 difference (24.51 cm for both RayStation MC and PBCS versus 24.89 cm measured). Further down the distal dose falloff, discrepancies decreased to approximately 0.2 cm, with an R20 difference of 0.17 cm for RayStation MC (25.06 cm vs. 25.23 cm measured) and 0.20 cm for RayStation PBCS (25.03 cm vs. 25.23 cm measured). These findings indicate strong agreement despite the complex heterogeneous setup, with potential for additional improvement through simplified positioning strategies, such as placing the Tough Water on top of the phantom to avoid repeated repositioning.

Focusing on R80, which is the most clinically relevant range parameter for commissioning, the percentage differences between calculated (MC and PBCS) and measured values (Table 2) were smaller than anticipated. Zheng et al.^16^ reported proton range differences exceeding 2% (up to 2.5%) and a range uncertainty of ∼1.5 mm ± 1.5 mm using film dosimetry in animal tissue with a single‐energy CT scanner (GE LightSpeed RT16; GE Healthcare, Wauwatosa, WI). In contrast, this study observed R80 differences within 1% for all beam paths except the lateral heterogeneity case (< 1.25%). This improved agreement is likely attributable to the use of multi‐energy CT; Zheng et al.^16^ used single‐energy CT, while this study employed a multi‐energy Philips IQon Spectral CT. Hua et al.^17^ and Schaeffer et al.^18^ demonstrated high accuracy and reproducibility of effective atomic number and electron density with dual‐energy CT, and Mohler et al.^19^ reported significantly lower mean absolute error for dual‐energy versus single‐energy CT using stoichiometric calibration (0.10 ± 0.15% vs 1.27 ± 0.12%), confirming reduced range uncertainty in biological tissue. These findings support the ability of multi‐energy CT to achieve low proton range uncertainty.

RayStation's MC and PBCS algorithms yield nearly identical calculated range parameter values for R90, R80, R50, and R20. This indicates that both algorithms handle proton range in a very similar fashion. However, prior studies report more pronounced differences in range and associated parameters, including dose coverage dependent on proton range cutoff, when comparing algorithms.^20^ Further investigation in more complex mixtures, such as thicker bone sections and/or lateral bone–tissue heterogeneities exceeding 3 cm, as well as inclusion of lung tissue, may reveal additional discrepancies.

## 5 CONCLUSION

Proton range measurements from distal falloff assessment using a common, easy‐to‐use ion‐chamber array closely align with both RayStation MC and PBCS algorithms. This strong depth‐dose curve and range parameter agreement between calculated and measured data validates the recently commissioned CT calibration curve and TPS for safe clinical use following experimental verification with animal tissue. The methodology presented provides a framework for validating the CT bilinear calibration curve used in clinical proton dose calculations, while avoiding cumbersome multilayer ionization chambers (MLICs).

## AUTHOR CONTRIBUTIONS

All authors contributed to the experimental design of the study. Andrew J. Boria, Chia‐Ho Hua, and Chin‐Cheng Chen performed data collection. All authors participated in data analysis, interpretation, and contributed to writing, revising, and editing the manuscript. All authors approve the final content and agree to be accountable for all aspects of the work.

## CONFLICT OF INTEREST STATEMENT

The authors declare no conflicts of interest.

## ETHICS STATEMENT

This study did not involve human participants or animals, and therefore ethical approval and informed consent were not required.

## Data Availability

The data supporting the findings of this study are available from the corresponding author upon reasonable request.
